# An Unusual Case of *Francisella tularensis*

**DOI:** 10.1155/2022/7250294

**Published:** 2022-04-18

**Authors:** Samantha Huang, Bradley Kaptur, Julius Manu, Elias Woldegabriel

**Affiliations:** ^1^Carle Illinois College of Medicine, University of Illinois Urbana-Champaign, Champaign, IL, USA; ^2^Department of Internal Medicine, Carle Foundation Hospital, Urbana, IL, USA; ^3^Department of Infectious Disease, Carle Foundation Hospital, Urbana, IL, USA

## Abstract

A 67-year-old male presented with complaints of weakness, fatigue, and shortness of breath in the context of a recent hospitalization for the same unresolved symptoms. After a largely nonspecific clinical presentation, a chest X-ray revealed a loculated pleural effusion. Culture of the postthoracentesis exudate revealed the culprit to be the aerobic Gram-negative bacterium *Francisella tularensis*. Amidst reports of potential resurgence, clinicians should be aware of the possible presentations of tularemia and consider it in the case of an ostensibly contributory patient history.

## 1. Introduction

The aerobic Gram-negative bacterium *Francisella tularensis* is responsible for the disease tularemia. Also known as “rabbit fever” or “wild hare disease,” tularemia is classically considered a zoonotic infection transmitted by ticks or various species of small- to medium-sized land mammals [1, 2]. It is considered a rare disease although there have been recent suggestions of reemergence in the literature, particularly in the European countries [3].

Tularemia has a variety of clinical syndromes that correspond to the portion of the body that is predominantly affected (i.e., ulceroglandular, glandular, oculoglandular, oropharyngeal, or pneumonic) [4]. Typhoidal tularemia refers to a systemic febrile disease that may begin with any portal of entry [4].

*Francisella tularensis* is highly virulent, with only inoculation with 10 to 50 organisms required to manifest with the clinical syndrome [5]. Interestingly, the organism is classified as a category A bioterrorism agent by the United States Centers for Disease Control and Prevention due to its suitability for use in targeted bioterrorism [6]; it has similar designations in other countries [7].

A high index of clinical suspicion for tularemia is needed due to the lack of specific clinical manifestations of the disease [8]. A history of known contact with high-risk animals is a key factor to justify the addition of tularemia to a working list of differential diagnoses [9].

## 2. Case Presentation

A 67-year-old male presented to our emergency department with complaints of weakness, fatigue, and shortness of breath over a week. He reported that his symptoms had started abruptly without any exacerbating cause. He had not had any sputum production, fevers, chills, night sweats, or sick contacts. He had a relevant past medical history of heart failure with reduced ejection fraction, persistent atrial fibrillation, essential hypertension, and obstructive sleep apnea. He had recently been seen at a smaller regional hospital for similar symptoms and discharged with adjustments to his heart failure medications including digoxin, furosemide, and metoprolol. On follow-up with his primary care physician, he was found to have low blood pressure and was promptly referred to the emergency department.

**Figure 1:**
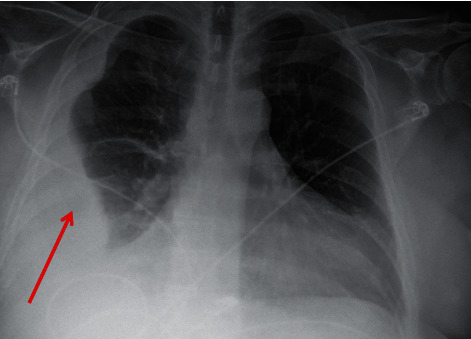
Chest X-ray of the patient upon admission demonstrating right-sided loculated pleural effusion (red arrow).

**Figure 2:**
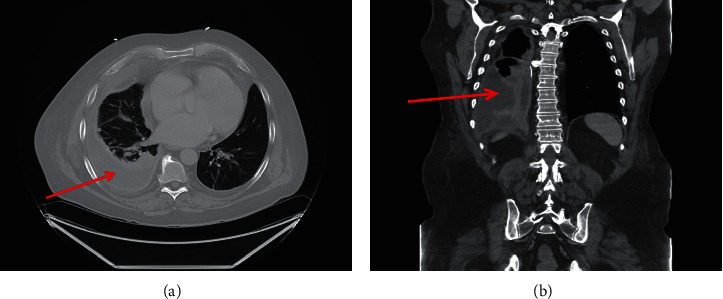
CT chest with contrast of the patient taken shortly after admission demonstrating pleural effusion in the (a) axial and (b) coronal views. The red arrow denotes pleural effusion.

On arrival, his blood pressure readings were in the 70 s/50 s·mmHg with EKG demonstrating atrial fibrillation with rapid ventricular response and occasional premature ventricular contractions. Chest X-ray revealed a loculated right-sided pleural effusion (Figure 1). His initial laboratory findings showed a white blood cell count of 13,200/mm^3^ (4,000–11,000/mm^3^) with absolute neutrophils of 11,200/mm^3^ (2,500–7,000/mm^3^). Serum sodium and potassium levels were 125 mEq/L (135–145 mEq/L) and 4.0 mEq/L (3.6–5.2 mEq/L), respectively. Transaminases were elevated at an AST of 289 units/L (<40 units/L) and an ALT of 228 units/L (<55 units/L). His Troponin I level was nonsignificant (<0.04 ng/mL), and his BNP was 46 pg/mL (<100 pg/mL). A CT scan (Figure 2) was performed during the workup, which demonstrated findings similar to the initial chest X-ray.

**Figure 3:**
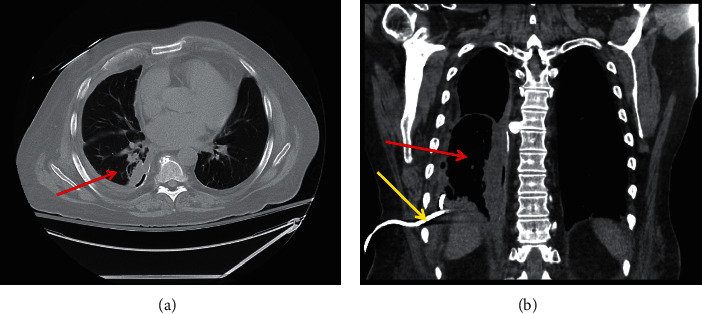
CT chest of the patient after chest tube placement in the (a) axial and (b) coronal views, suggesting progressive resolution. The red arrow denotes resolving pleural effusion. The yellow arrow denotes the chest tube.

After a consultation with Interventional Radiology, the decision was made to drain the pleural effusion. A thoracentesis was performed that yielded a leukocyte-dominant exudate. Cytology revealed no malignant cells. Postprocedure chest X-ray revealed improved, but persistent, loculated effusions and a subpleural pulmonary nodule. An IR ultrasound/fluoroscopy-guided chest tube was placed that drained 3 liters of serosanguinous fluid. Repeat CT chest (Figure 3) was performed 3 days after placement of the chest tube.

Preliminary pleural fluid aerobic culture was suggestive of *Francisella tularensis*, and the sample was sent to the Illinois Department of Public Health, which later confirmed the identified pathogen.

The patient had been previously started on ertapenem 1 gram IV daily for suspected empyema. Upon receipt of the culture results, tobramycin was added and ertapenem was subsequently discontinued, based on the recommendations of the Infectious Disease consultant. 10 doses of tobramycin were planned with dosing per pharmacist recommendation, and the patient was discharged with the intent to return for his 2 remaining doses.

## 3. Discussion

*Francisella tularensis* has been the causative organism in both primary and secondary pneumonic diseases, which are differentiated by direct inhalation and hematogenous spread, respectively. Clinical presentations dominated by pulmonary involvement are termed pneumonic tularemia. Previous case reports have described instances of tularemia presenting as an isolated pleural effusion [10].

In our case, the patient was unable to identify a specific incident of likely exposure although his occupation as a mailman may have placed him at a higher risk. It has been previously demonstrated that patients with pneumonic disease were more likely to recall no potential exposure [11]. The patient resided within the referral range of our hospital, which covers the majority of central Illinois. This is on the periphery of the *Francisella tularensis* hotspot that centers in the region of Missouri and Arkansas, as reported by the CDC [5]. Our patient frequently engaged in outdoor activities such as mowing his large lawn. He also had a career that required him to frequently traverse wooded and fielded areas throughout rural central Illinois. It is likely he was exposed to the organism during one of those activities although it is not possible to conclude this definitively.

Cases in North America tend to be reported during the summer and fall seasons [12], and this was the case with our patient as well. Tularemia may be a differential diagnosis suspected when a patient presents with fever and lymphadenopathy in conjunction with known contact with animals such as rabbits, deer, or ticks [9]; however, even after discussing the diagnosis with our patient, he was still unable to pinpoint such an exposure.

Laboratory findings in tularemia tend to be nonspecific [13, 14] but may include elevated white blood cell count, low serum sodium, and abnormal liver enzymes, all of which were seen in our patient. Myoglobinuria or evidence of rhabdomyolysis may be seen, but fortunately, that was not the case in our patient [15].

Antimicrobial therapy in the form of an aminoglycoside has traditionally been the preferred treatment in the United States, with streptomycin and gentamicin being preferred agents [16]. Other acceptable antibiotic choices include fluoroquinolones and tetracyclines. In our patient, the aminoglycoside tobramycin was chosen for its less drug-resistant profile in the context of limited antibiotic sensitivity testing capabilities. The concern surrounding antibiotic-resistant strains has motivated research into novel therapeutic strategies, including new inhibitory molecules (galantamine, polyinosine-polycytosine, etc.) in isolation or combination therapy [17]. There was also some concern for aminoglycoside penetration into the pleural cavity in our patient, but subsequent radiologic imaging documenting improvement suggested that the therapy was effective.

## 4. Conclusion

Tularemia remains a fairly rare diagnosis, with exudative pleural effusion being a possible presentation. Gentamicin is traditionally a preferred treatment agent although tobramycin is an acceptable choice as well.

## Data Availability

No data were used to support this study.
